# “Going above and beyond” for implementation in the education sector: extension and validation of the School Implementation Citizenship Behavior Scale (SICBS)

**DOI:** 10.1186/s43058-025-00748-3

**Published:** 2025-05-26

**Authors:** Catherine M. Corbin, Mark G. Ehrhart, Eric C. Brown, Jill Locke, Clayton R. Cook, Gregory A. Aarons, Aaron R. Lyon

**Affiliations:** 1 School of Special Education, School Psychology, and Early Childhood Studies, University of Florida, Norman Hall Rm. 1801, Gainesville, FL32611 1801 USA; 2 Department of Psychology, University of Central Florida, Orlando, FL USA; 3 Department of Public Health Sciences, Miller School of Medicine, University of Miami, Miami, FL USA; 4 Department of Psychiatry and Behavioral Sciences, University of Washington, Seattle, WA USA; 5 Character Strong, Puyallup, WA USA; 6 Department of Psychiatry, University of California, San Diego, USA; 7 ACTRI Dissemination and Implementation Science Center, UC San Diego, San Diego, CA USA; 8 Child and Adolescent Services Research Center, San Diego, CA USA

**Keywords:** Implementation citizenship behavior, Measurement, Education, Prevention, Mental Health

## Abstract

**Background:**

Employee behaviors that strategically support implementation (i.e., implementation citizenship behavior [ICB]) theoretically promote the adoption and high-fidelity use of evidence-based practices (EBPs). ICB (e.g., helping colleagues overcome implementation barriers) may vary across contexts, including schools where children are most likely to access and receive mental and behavioral health services. Pragmatic measures are needed to advance nascent research on school-based ICB and inform how these behaviors can be used to support successful implementation. The current study expanded the Implementation Citizenship Behavior Scale (ICBS) to create and validate the School Implementation Citizenship Behavior Scale (SICBS) in a sample of elementary school teachers implementing evidence-based prevention programs to support children’s mental and behavioral health.

**Methods:**

Based on subject matter expert feedback, items were refined from the original ICBS and items for two new subscales (taking initiative, advocacy) were created for the SICBS. A sample of 441 public school teachers from 52 elementary schools in the Midwest and Western United States of America completed a survey that included the SICBS and additional measures to assess convergent and divergent validity. SICBS was refined and validated via examination of item characteristics curves to reduce items and develop a pragmatic instrument, confirmatory factor analyses to evaluate the hypothesized measurement structure, and assessment of convergent and divergent validity.

**Results:**

The original two ICBS subscales (helping others, keeping informed) were retained, and two new three-item subscales resulted from item reduction analyses (taking initiative, advocacy). The hypothesized second-order factor model was generally well fit to the data (CFI = .99, TLI = .99, RMSEA = .09), all first- (λs = .85-.96) and second-order factor loadings (λs = .93-.95) were high. All SICBS subscales demonstrated acceptable reliability (αs = .88-.92). Convergent validity was evidenced by moderate correlations with organizational citizenship behavior items (*r*s = .42-.49). Divergent validity was demonstrated by weak correlations with teachers’ beliefs about teaching (*r*s = .31-.38) and null correlations with most school demographics.

**Conclusion:**

Results support the structural, convergent, and divergent validity of the 12-item, 4-factor SICBS. The SICBS provides a deeper understanding of individual implementer actions that may serve as implementation mechanisms or outcomes.

**Supplementary Information:**

The online version contains supplementary material available at 10.1186/s43058-025-00748-3.

Contributions to the literature

- Employees’ prosocial behavior that strategically supports evidence-based practice (EBP) implementation (i.e., implementation citizenship behavior), like helping colleagues solve implementation-related challenges, may improve implementation quality across schools, where most children and youth access and receive mental and behavioral health services.
- This study expands the Implementation Citizenship Behavior Scale to create and validate the School Implementation Citizenship Behavior Scale among teachers implementing evidence-based prevention programs to support children’s mental and behavioral health.
- Findings supported the two original in addition to two new subscales, resulting in the four-factor School Implementation Citizenship Behavior Scale, which demonstrated convergent and divergent validity.

## Background

The adoption, high-fidelity use, and sustainment of evidence-based practices (EBPs) are critical to preventing and addressing growing mental and behavioral health concerns among youth [19, 56, 69]. A substantial body of work has identified factors likely to impact implementation effectiveness across service settings [13, 30, 46], including schools where youth are most likely to access and receive preventive and indicated mental health services [18, 32]. Aspects of the organizational context are particularly important determinants (i.e., barriers and facilitators) of implementers’ routine high-quality use of EBP [2, 34]. Although considerable research has focused on the role of leadership and organizational climate (e.g., [43, 45, 71, 72]), fewer studies have addressed the role employees and their peers play in facilitating implementation [25]. Further, the limited availability of validated assessment instruments of these influences has inhibited implementation research and practice in the school context. The development of the Implementation Citizenship Behavior Scale (ICBS [20]) and its school-adapted version [39] provide an opportunity to uncover how employee behavior might be cultivated to strategically support EBP implementation. This study extends the work of Ehrhart et al. [20] and Lyon et al. [39] by validating the School Implementation Citizenship Behavior Scale (SICBS)—adapted to include two new subscales (taking initiative and advocacy)—among teachers implementing evidence-based universal (i.e., delivered to all students) school-based prevention programs.

### Organizational citizenship behavior and EBP implementation

Organizational citizenship behavior (OCB) describes “individual behavior that is discretionary, not directly or explicitly recognized by the formal reward system, and in the aggregate promotes the efficient and effective functioning of the organization” [50], p. 3). Simply put, OCB describes activities by employees that go “above and beyond” their typical assigned duties to advance broader organizational goals. Specific behaviors underlying OCB can be placed into two major categories: OCB directed toward individuals (e.g., helping or courtesy) or OCB directed towards the organization (e.g., voice or civic virtue) [70]. Predictors of OCB cut across characteristics of individuals (e.g., personality or attitudes), the task (e.g., feedback), the organization (e.g., organizational structure, cohesiveness), and leaders (e.g., reward behaviors, support) [51]. Moreover, increased OCB among individuals and groups predicts a range of multilevel indicators of organizational effectiveness including employee performance, decreased turnover, team effectiveness, and healthcare outcomes [52, 53].

One development in the OCB literature is the consideration of strategic forms of OCB, or in other words, employee behaviors that exceed expectations in support of a prioritized goal or initiative. For example, customer-focused OCB is associated positively with customer satisfaction [61], and similar findings have emerged related to safety-focused OCB [24]. An emerging body of research has extended strategic OCB to include a focus on EBP implementation; implementation citizenship behavior (ICB) describes how employees support effective implementation across an organization [20]. Implementers demonstrate ICB via prosocial behaviors that support colleagues’ adoption and high-fidelity use of EBP and by investing personal resources to remain abreast of changes to and organizational communication about EBP [20, 39].

Pragmatic (i.e., comprehensive, brief, useful) measures [22] of implementation-related behaviors are needed to advance understanding of how they might be leveraged to address the research-to-practice gap. The Implementation Citizenship Behavior Scale (ICBS; [20]) is one such measure that has been validated for use in mental health clinics in the United States and Norway [8, 20] and has demonstrated positive associations with EBP attitudes, clinicians’ intentions to implement EBPs, employee- and supervisor-rated performance, and employee tenure [8, 20, 67]. The original ICBS dimensions and items also have been adapted and validated for use among school-based mental and behavioral health consultants [39], though not among frontline implementers of school-based universal mental and behavioral prevention programs, such as teachers. In addition, this past work simply changed the referent of the original ICBS items to be appropriate for school settings but did not obtain subject matter expert input on the appropriateness of the items for school settings or on the possibility of additional dimensions that would be relevant for implementation in schools. There are a variety of dimensions of OCB that have been identified in that literature [51]; the dimensions included in the ICBS were simply those identified to be most critical in mental health settings. Thus, the next step for research in this area is to use a more rigorous process to validate and possibly expand the existing measure, in line with the suggestion by the developers of the school-adapted ICBS that the measure could be expanded to capture additional extra-role behaviors integral to supporting effective EBP implementation in school settings [39]. 

### School-based EBP and implementation citizenship behavior

 Schools provide a continuum of mental and behavioral healthcare to youth, ranging from primary prevention (i.e., universal) to more intensive tiers of service provision (i.e., indicated, delivered individually or to small groups of students) [4]. Universal prevention programs, like mental health literacy [41] or social-emotional learning interventions [9], are critical as they can interrupt and/or reverse maladaptive developmental trajectories at a point when they may be more readily influenced [63]. The continuous rise in mental health conditions among youth over the past few years [29, 56] clearly underscores the need to identify and develop proximal resources to support effective school-based EBP implementation.

Individual implementer characteristics routinely emerge as integral to successful EBP implementation [55]. Despite a dearth of research linking ICB to implementation outcomes, related constructs suggest the potential of ICB to positively influence EBP implementation. For example, opinion leaders—employees who disproportionately influence the attitudes and behaviors of their peers [59]—engage in behaviors that overlap with ICB, like sharing resources to support colleagues’ implementation or communicating the benefits of EBP. Key opinion leaders demonstrating these kinds of behaviors have been shown to support classroom teachers’ use of evidence-based practices for students with ADHD [5].

In addition to the original dimensions of the ICBS (helping and keeping informed), focus groups with educational stakeholders (e.g., principals, teachers) have indicated there may also be additional extra-role implementation-related behaviors teachers practice that are specific to the school implementation context (e.g., volunteering to observe or be observed; working with colleagues, families, and community members to promote EBP implementation) [37]. Based on that work and the additional feedback outlined in the Method section, two new ICBS subscales are proposed to measure the extent to which teachers (a) take initiative by participating in EBP related activities (e.g., supplemental trainings, being observed by other implementers) that directly support implementation and (b) actively promote (e.g., share positive benefits of EBP) and advocate for EBP use in interactions with peers and other stakeholders (i.e., advocacy). Notably, these dimensions align with existing frameworks of OCB dimensions, including taking initiative and the individual initiative dimension of OCB (i.e., voluntarily taking on extra responsibilities)and advocacy and the organizational loyalty (i.e., externally promoting the organization) dimension [51]. Thus, just as the development of the original ICBS was informed by OCB theory and research [20], the alignment of these additional dimensions with past OCB literature gives credence for the consideration of taking initiative and advocacy as potential components of ICB.

### Study aims

As indicated in multiple implementation frameworks [13, 46], identifying and validly measuring characteristics of the organizational context likely to improve EBP implementation is imperative for research and practice. This study expands upon behaviors measured by the original ICBS by adding two new subscales and validating the SICBS among elementary school teachers implementing one of two universal EBPs: Schoolwide Positive Behavioral Interventions and Supports (SWPBIS; [33] and Promoting Alternative THinking Strategies (PATHS, [23]). SWPBIS is a strategy that promotes desired behavior change among students via systems-level change (e.g., altering discipline practices), data-driven decision-making, and selection of EBPs aligned with student need [64]. PATHS is a classroom-based curriculum that includes lessons to promote students’ self-regulation, emotion-regulation, and positive peer interactions [28]. Confirmatory factor analysis was used to establish the measurement structure of the adapted SICBS, followed by item response theory to refine newly added subscales. Convergent and divergent validity were established using data on teachers’ OCB, attitudes toward teaching, and school demographics. Moreover, recent work validating related instruments tested invariance across versions that referenced either a specific EBP or EBP in general [40, 66] to determine whether items with more specificity (i.e., specific EBP, which may more strongly predict implementation outcomes), measured the same underlying construct as the more general items. Aligned with these efforts, we also examined the invariance of the SICBS across survey referents.

We hypothesized the following:

- *Hypothesis 1:* The adapted SICBS with a second-order factor structure will fit the data such that four first order factors (helping others, keeping informed, taking initiative, advocacy) will comprise one higher order School Implementation Citizenship Behavior factor.
- *Hypothesis 2: *Correlations between the adapted SICBS and OCB total and subscale scores will be moderate to strong (convergent validity).
- *Hypothesis 3*: Correlations between the adapted SICBS total and subscale scores, teachers’ general attitudes toward teaching, and school demographics will be weak and/or nonsignificant (divergent validity).
- *Hypothesis 4: *The SICBS will be invariant across EBP referent type (i.e., general vs. specific).

## Method

### Setting and participants

#### Setting

Schools were eligible to participate if implementing one of two evidence-based interventions (*n* = 39 SW-PBIS; *n* = 13 PATHS). A total of 441 teachers from 52 elementary schools in 6 school districts in Washington, Ohio, and Illinois were recruited. On average, students across schools were 66% non-White (range = 21 to 100%) and 57% low-income status (range = 4 to 100%). Of the 500 teachers contacted to participate, 88% completed the online survey (see Procedures).

#### Teacher-level demographics

An average of nine teachers per school were invited to complete the survey. Most teachers were female, White, reported an average of 11.6 years of experience, and held a master’s degree (Table 1). The analytic sample was sometimes less than 441 due to missing data (< 5% across all study constructs).

**Table 1:** Participant demographics for School Implementation Citizenship Behavior Scale (SICBS) General (*N* = 218), Specific (*N* = 222), and combined (*N* = 440) samples

Participant Information	General Freq (%)	Specific Freq (%)	Combined Freq (%)
Age			
18 to 24 years old	7 (3.2)	14 (6.3)	21 (4.8)
25 to 34 years old	65 (30.0)	64 (29.0)	129 (29.5)
35 to 44 years old	58 (26.7)	63 (28.5)	121 (27.6)
45 to 54 years old	55 (25.3)	47 (21.3)	102 (23.3)
55 to 64 years old	31 (14.3)	30 (13.6)	61 (13.9)
65 to 74 years old	1 (0.5)	3 (1.4)	4 (0.9)
Total	217 (100.0)	221 (100.0)	438 (100.0)
Gender			
Male	26 (12.0)	19 (8.6)	45 (10.3)
Female	190 (87.6)	201 (91.4)	391 (89.5)
Other	1 (0.5)	0 (0.0)	1 (0.2)
Total	217 (100.0)	220 (100.0)	437 (100.0)
Race			
American Indian or Alaskan Native	7 (3.2)	1 (0.5)	8 (1.9)
Asian	1 (0.5)	5 (2.3)	6 (1.4)
Black or African American	14 (6.5)	8 (3.7)	22 (5.1)
Native Hawaiian or Pacific Islander	0 (0.0)	1 (0.5)	1 (0.2)
White or Caucasian	178 (82.4)	184 (85.2)	362 (83.8)
Multiracial	11 (5.1)	10 (4.6)	21 (4.9)
Other	5 (2.3)	7 (3.2)	12 (2.8)
Total	216 (100.0)	216 (100.0)	432 (100.0)
Ethnicity			
Latino/Hispanic	14 (6.5)	17 (7.7)	31 (7.1)
Non-Latino/Hispanic	203 (93.5)	203 (92.3)	406 (92.9)
Total	217 (100.0)	220 (100.0)	437 (100.0)
Highest Degree Earned			
Bachelors	72 (33.2)	68 (30.9)	140 (32.0)
Masters	144 (66.4)	152 (69.1)	296 (67.7)
Doctoral	1 (0.5)	0 (0.0)	1 (0.2)
Total	217 (100.0)	220 (100.0)	437 (100.0)
Grade			
K – 2nd	91 (41.7)	99 (44.6)	190 (43.2)
3rd – 5 th and other	127 (58.3)	123 (55.4)	250 (56.8)
Total	218 (100.0)	222 (100.0)	440 (100.0)
	PBIS T1 N, Mean ± sd	PATHS N, Mean ± sd	COMBINED N, Mean ± sd
Years in Current Role	217, 11.9 ± 6.9	220, 11.3 ± 7.1	437, 11.6 ± 7.0
Years at Current School	217, 7.0 ± 6.0	220, 6.9 ± 5.9	437, 6.9 ± 6.0

### Procedures

This study was part of a federally-funded measure adaptation and development project that produced validated assessments of the school organizational implementation context. Feedback from focus groups with educator stakeholder groups [37] informed the initial modification of the school-adapted ICBS [39]. Changes were made to item wording to ensure construct equivalence for school-based implementers [26]. The current study further expanded the original two dimensions of the school-adapted ICBS of helping and keeping informed to include items that measure behavioral indicators hypothesized to underlie the extent to which teachers supported EBP implementation by (a) taking initiative and engaging in (b) advocacy. The details of this process are provided in the description of the SICBS in the Measures section.

Human subjects approval was obtained from the University of Washington Institutional Review Board and, when applicable, participating school districts’ research and evaluation departments. Recruitment began with study investigators contacting school district central administrators to discuss the project and secure participation. Participating central administrators then identified eligible schools and helped distribute information about the study, its benefits, and data collection procedures to site-based administrators. The opportunity to participate was presented to teachers, typically during staff meetings or via email, resulting in the recruitment of 4–10 teachers from each school. Research staff used contact information from participating teachers for all project communications (e.g., sending survey links).

Data were collected between September and November of 2017. Initial email communication to teachers provided a project description, obtained informed consent, and included a unique link to the web-based survey. Teachers had one-month from receipt of the initial email to complete their survey. Weekly email reminders were sent in an effort to increase survey response rates.

### Measures

#### School Implementation Citizenship Behavior (SICBS)

The original ICBS [20] and initial school-adapted ICBS [39] are six-item instruments designed to measure implementer behaviors strategically directed toward effective EBP implementation. The ICBS is intended to be completed by supervisors and the school version by individual implementers, thus measuring slightly different forms of ICB. All items are scored on a 4-point Likert scale that ranges from 0 (*not at all*) to 4 (*to a very great extent*). Past research has supported the measurement structure of both instruments as characterized by two correlated latent factors (helping others, keeping informed) defined by three items each [20, 39]. The exploration of whether additional dimensions were needed to capture important dimensions of implementation citizenship in educational settings occurred across two phases: focus groups with subject matter experts and a follow-up expert summit. In both phases, experts were provided with the definition of implementation citizenship and the existing ICBS measure with school-appropriate referent (e.g., schools, teachers). They then provided feedback on the appropriateness of the existing dimensions and items, as well as on the need for additional dimensions. Educational stakeholders (e.g., teachers, principals) in the initial focus groups first provided feedback indicating the need for additional school-based ICB dimensions, which were subsequently refined and organized by expert summit participants and the research team into taking initiative (*n* = 4 candidate items) and advocacy (*n* = 5 candidate items) (see Table 2 for list of new candidate items). Combining these items with the six adapted versions of the original items, the initial version of the adapted SICBS included 15 items. Item reduction procedures, reliability, and validity of the adapted SICBS are reported in the Results. Additionally, and consistent with other instruments developed in the context of the broader project [40, 66], two versions of the adapted SICBS were created that differed in the referent used. Items in one version referenced EBP generally (e.g., “*School staff keep informed of changes in EBPs*”), whereas items the other version referenced a specific EBP (e.g., “*School staff keep informed of changes in SWPBIS/PATHS*”). Invariance in the underlying factor structure of the EBP-general and EBP-specific versions of the adapted SICBS was tested using multigroup modeling (see Results).

**Table 2:** Candidate items considered for inclusion in the two new School Implementation Citizenship Behavior Scale subscales

Subscale	Candidate Item
Taking Initiative	7. Teachers/school staff take initiative surrounding the implementation of EBP 8. Teachers/school staff feel responsible for the implementation of EBP for the greater good of the school 9. Teachers/school staff participate in voluntary activities (e.g., extra meetings) involving the implementation of EBP 10. Teachers/school staff willingly take on additional responsibilities related to the implementation of EBP
Advocacy	11. Teachers/school staff promote the importance of EBP when communicating with individuals outside of the school (e.g., parents) 12. Teachers/school staff advocate for EBP implementation in their interactions with other staff 13. Teachers/school staff work to ensure that staff see the positive benefits of EBP implementation 14. Teachers/school staff defend the use of EBP to improve student outcomes 15. Teachers/school staff here are proponents of EBP

#### Organizational Citizenship Behavior in Schools (OCBS)

The OCBS [17] is a 12-item scale that measures teachers’ perceptions of general (i.e., not implementation-specific) OCB among colleagues in their school. Teachers rated their agreement with items using a 4-point Likert scale ranging from 1 (*Strongly disagree*) to 4 (*Strongly agree*). The OCBS captures behaviors directed toward individuals (e.g., “*Teachers/school staff voluntarily help new teachers*”) and the organization (e.g., “*Teachers/school staff committees in this school work productively*”). Evidence supports OCBS as a unitary construct with strong psychometric properties [16]. Internal consistency was acceptable (α = 0.75).

#### Public School Teacher Questionnaire (PSTQ)

The PSTQ—a long-time staple of the Schools and Staffing Survey conducted by the National Center for Education Statistics [48]—is a measure of teachers’ general attitudes toward teaching. We anticipated teachers’ general attitudes would correlate weakly with a specific type teaching-adjacent behavior (i.e., implementation citizenship) and thus included the PSTQ as a measure of divergent validity. Teachers responded to nine items assessing various attitudes toward the teaching profession (e.g., “*The teaching profession is something that I enjoy and feel competent doing*”) using 4-point Likert scale ranging from *strongly disagree* to *strongly agree*. The PTSQ is a psychometrically validated scale [58] and demonstrated acceptable internal consistency in this sample (α = 0.81).

### Data analytic approach

Confirmatory factor analysis (CFA) was used to assess the extent to which each hypothesized latent factor is comprised by the proposed subscale items. SICBS subscale ICCs indicated a substantial amount (18–22%) of between-school variance. Therefore, CFAs were tested in *Mplus* v8 [47] specifying robust standard errors and using type = complex to account for the nested structure of the data and weighted least squares means and variances (WLSMV) estimation with delta parameterization given the ordered-categorical response scale. Model fit was assessed using the following indices and associated thresholds as evidence of a model well fit to the data: chi-square test statistic, Comparative Fit Index (CFI ≤ 0.95; [27]), Tucker-Lewis index (TLI ≤ 0.95 [68],), the root mean square error of approximation (RMSEA ≤ 0.05,[7, 57]), and the standardized root mean residual (SRMR ≤ 0.08; [27]). Standardized factor loadings (*λ*) less than 0.55 were considered low and required further examination [65]. The minimum sample size to detect close to exact model fit based on RMSEA with 80% power is *n* = 221 [42].

Two measurement models were examined and compared. The first included four correlated latent factors representing the hypothesized SICBS subscales. The second model specified a second-order latent factor in which the four first-order factors loaded onto the higher-order Implementation Citizenship Behavior factor. Each of these models was tested once prior to and once post item reduction. Guided by theory and aligned with the goal of developing a comprehensive and pragmatic measure of school ICB, the decision was made to prioritize the second-order factor model if the four first-order latent factors demonstrated appreciable factor loadings.

To ensure the development of a pragmatic measure, we used item response theory (IRT) analysis to retain the three items that best represented each of the two newly added subscales [14]. IRT analyses provide information about how each subscale item functions along several dimensions including the amount of information each item contributes along the continuum of the school implementation citizenship behavior latent trait [73]. Pairing the unique information yielded from CFA and IRT analyses is a robust way to assess and refine subscales [6].Item characteristic curves were examined by the study team and decisions regarding item reduction were made to maximize item coverage and limit redundancy of information. After reducing items, we again tested both CFA models. Next, the invariance of the underlying factor structure of the EBP-general and EBP-specific versions of the adapted SICBS was tested using multigroup modeling. Two statistics were used in favor of the chi-square test statistic, which is heavily influenced by sample size [44]. The *q* effect size was used to estimate the difference in magnitude between factor loadings across survey type, where values that cluster around zero indicate no substantive difference and values of 0.10, 0.30, and 0.50 indicate a small, medium, and large effect size, respectively [54]. Additionally, *d*_Cox_, which ranges from 0 to 1 [60], was used to estimate the between-group differences in thresholds. In the absence of agreed upon cut points for *d*_Cox_, we used a decision rule informed by similar effect sizes [10] such that values above 0.50 indicated moderate differences and would be flagged for further investigation.

Convergent and divergent validity were assessed by examining correlations among SICBS total and subscale mean scores and those of measures that were hypothesized to yield moderate to strong (convergent) or weak to no (divergent) association. The SICBS is hypothesized to be a unitary construct and moderate-to-strong correlations among subscales were therefore anticipated. The OCBS—a general form of citizenship behavior—was expected to demonstrate moderate-to-large correlations with the SICBS (convergent validity). We hypothesized that teachers’ attitudes toward teaching would be weakly positively related to SICBS, and less so than global OCB (divergent validity). Finally, teachers’ experience implementing EBP may be influenced by certain school-level demographics. As such, null-to-weak correlations between SICBS total and subscale mean scores and school-level demographics were hypothesized.

## Results

### Preliminary confirmatory factor analysis

All fit indices except the RMSEA indicated acceptable fit for the first-order (CFI = 0.987, TLI = 0.98, RMSEA = 0.09, SRMR = 0.02) and the second-order factor models (CFI = 0.984, TLI = 0.98, RMSEA = 0.097). First-order factor loadings were all greater than 0.55 (λs = 0.84–0.96) and correlations among the four latent factors were high (*r*s = 0.86–0.93). Second-order factor loadings were appreciable (λs = 0.93–0.97). An RSMEA greater than 0.10 may be evidence of model misspecification. As a sensitivity check, we also examined a one-factor model in which all SICBS items loaded onto a single latent factor. This measurement model was prioritized over alternatives because of the high correlations among latent factors in the four-factor model (i.e., ≤ 0.86). The one-factor model demonstrated worse fit across all fit indices (CFI = 0.97, TLI = 0.96, RMSEA = 0.14, SRMR = 0.04) compared the four-factor first- and second-order models. Best practice when using structural equation modeling is to rely on theory to select models when statistical results are mixed. As such, we proceed with the second-order factor model.

### Item reduction

**Fig. 1:**
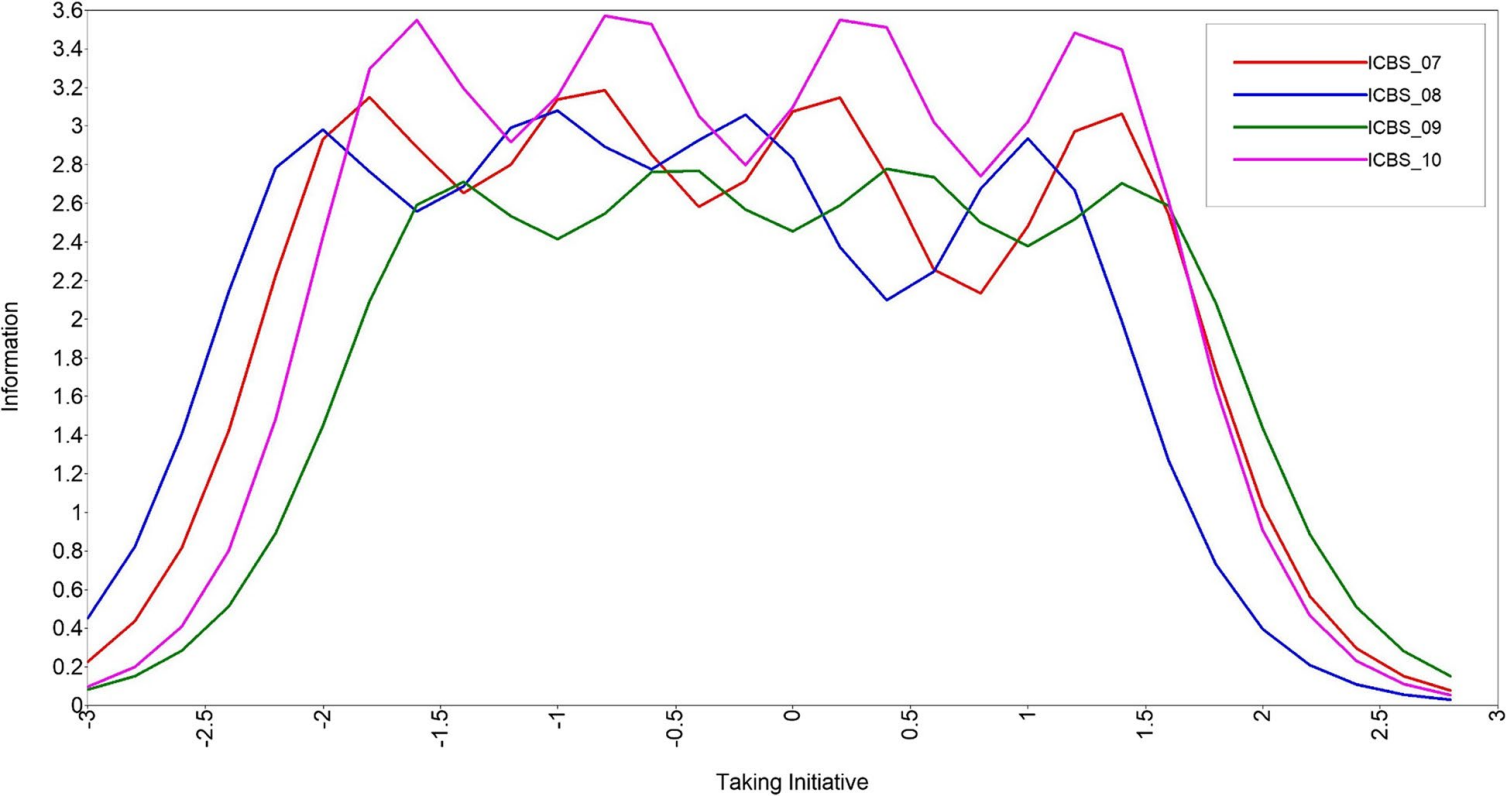
Information curves for candidate taking initiative subscale items

**Fig. 2:**
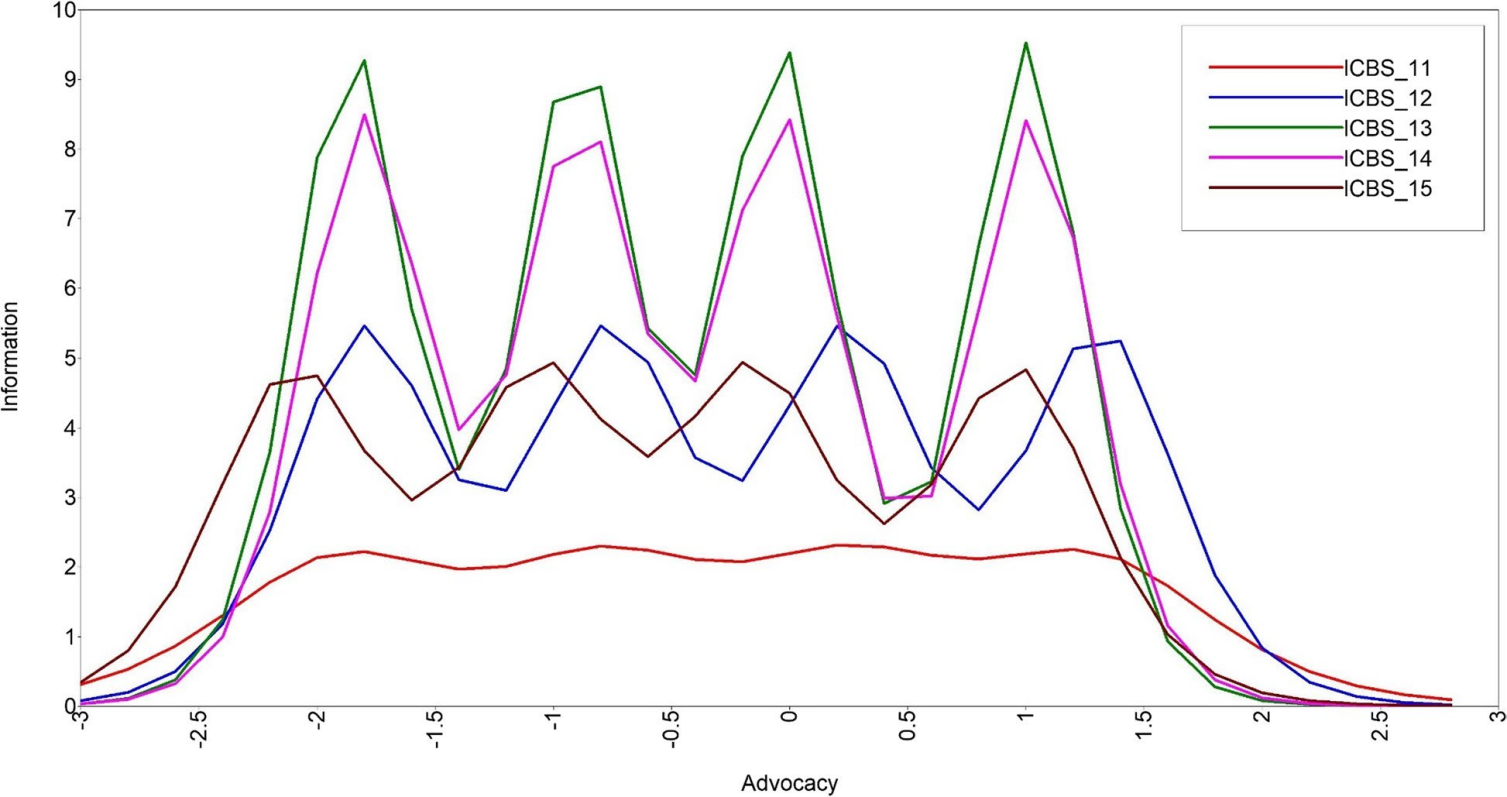
Information curves for candidate advocacy subscale items

Figures 1 and 2 present the item characteristic curves for the candidate items of the two new subscales: taking initiative (*n* = 4; items 7–10) and advocacy (*n* = 5; items 11–14). There was overlap of information provided by all taking initiative candidate items. Though the eighth item captures a small amount of unique information on the lower end of the taking initiative latent continuum (i.e., the curve is shifted to the left on the x-axis), the item was less behavioral than the other subscale candidate items. Therefore, Item 8 was dropped from the taking initiative subscale. Information provided across advocacy candidate items was more variable. Item 11 provided very little information (i.e., a consistently low peak across the x-axis). Items 12 and 14 provided a similar amount, but unique, information (i.e., peaks did not overlap). Conversely, Items 13 and 14 provided near identical information. Item 13 provided more information than Item 14 and the team preferred the wording and content of Item 13. For these reasons, Items 11 and 14 were dropped from the advocacy subscale. Table 3 presents summary statistics reliabilities for each SICBS subscale and Table 4 presents individual item response frequencies.

**Table 3:** Summary statistics for School Implementation Citizenship Behavior (SICBS) subscales

ICBS Subscale	*N*, *M* ± *SD*	Cronbach’s α
Helping Others	440, 2.20 ± 0.94	.91
Keeping Informed	440, 2.08 ± 0.99	.92
Taking Initiative	440, 2.16 ± 0.96	.88
Advocacy	440, 2.40 ± 0.96	.92

**Table 4:** Response frequencies for School Implementation Citizenship Behavior Scale (SICBS) items

SICBS Subscale	Not at All *n*, %	Slight Extent *n*,%	Moderate Extent *n*,%	Great Extent *n*,%	Very Great Extent *n*,%
Helping Others					
Teachers/school staff assist others to make sure they implement EBP properly	23, 5.2	78, 17.7	171, 38.9	125, 28.4	43, 9.8
Teachers/school staff help teach EBP implementation procedures to new staff	27, 6.1	88, 20.0	151, 34.3	132, 30.0	42, 9.5
Teachers/school staff help others with responsibilities related to the implementation of EBP	24, 5.5	75, 17.0	163, 37.0	136, 30.9	42, 9.5
Keeping Informed					
Teachers/school staff keep informed of changes in EBP policies and procedures	36, 8.2	87, 19.8	156, 35.5	124, 28.2	37, 8.4
Teachers/school staff keep up with the latest news regarding EBP	43, 9.8	111, 25.2	152, 34.5	103, 23.4	31, 7.0
Teachers/school staff keep up with school communication (e.g., memos, announcements, etc.) related to EBP	32, 7.3	69, 15.7	157, 35.7	134, 30.5	48, 10.9
Taking Initiative					
Teachers/school staff take initiative surrounding the implementation of EBP	22, 5.0	71, 16.1	150, 34.1	149, 33.9	48, 10.9
Teachers/school staff participate in voluntary activities (e.g., extra meetings) involving the implementation of EBP	42, 9.6	101, 23.1	153, 34.9	105, 24.0	37, 8.4
Teachers/school staff willingly take on additional responsibilities related to the implementation of EBP	30, 6.8	83, 18.9	153, 34.8	124, 28.2	50, 11.4
Advocacy					
Teachers/school staff advocate for EBP implementation in their interactions with other staff	22, 5.0	82, 18.6	159, 36.1	131, 29.8	46, 10.5
Teachers/school staff work to ensure that staff see the positive benefits of EBP implementation	18, 4.1	65, 14.8	130, 29.5	158, 35.9	69, 15.7
Teachers/school staff here are proponents of EBP	13, 3.0	57, 13.0	123, 28.0	170, 38.6	77, 17.5

### Confirmatory factor analysis post-item reduction

**Fig. 3:**
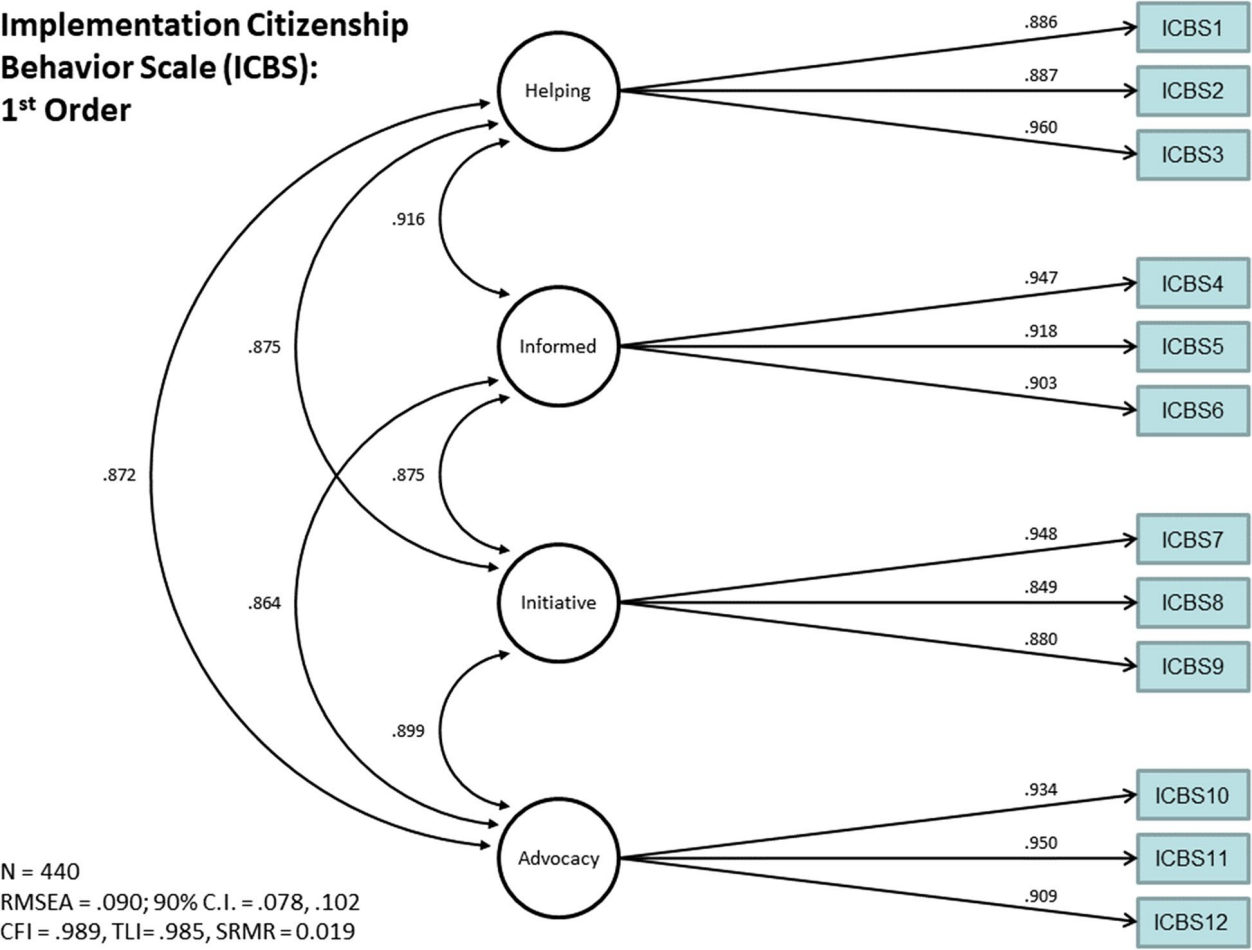
First-order school implementation citizenship behavior scale factor loadings

**Fig. 4:**
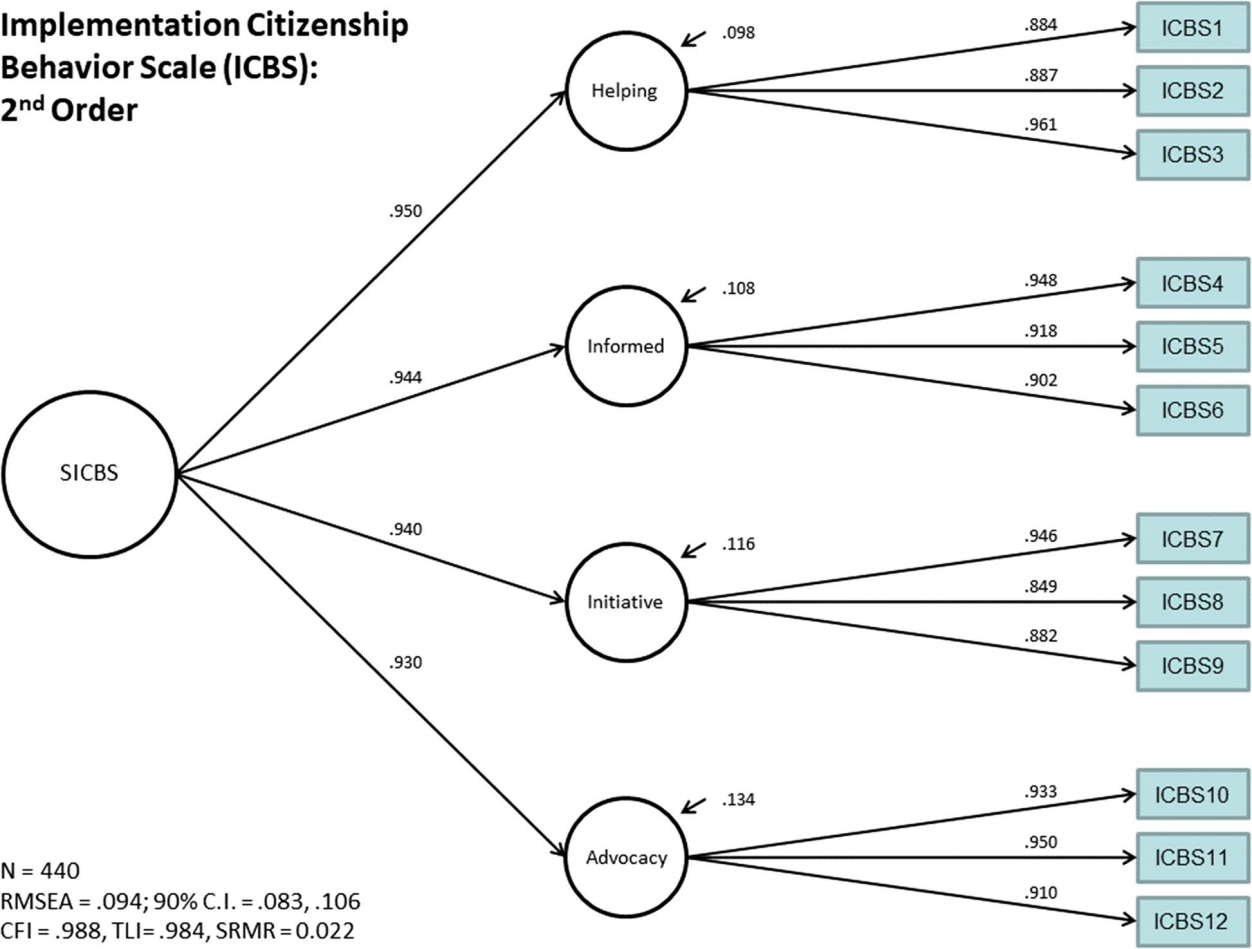
Second-order school implementation citizenship behavior scale factor loadings

The first- (Fig. 3) and second-order factor models (Fig. 4) were re-assessed using the reduced version of the SICBS. Both models again demonstrated identical and acceptable fit to the data (CFI = 0.99, TLI = 0.99, RMSEA = 0.09, SRMR = 0.02), though the RMSEA was above the desired threshold of 0.05. First-order factor loadings were high (λs = 0.85–0.96) and correlations among the first-order latent factors were high, *r*s = 0.86–0.92. Second-order factor loadings were well above the 0.55 threshold (λs = 0.93–0.95). Aligned with our goal of producing a pragmatic measure and aligned with theory and psychometric results, the second-order factor structure was retained.

### Measurement invariance

Two models (freely estimated, parameters constrained to equality) were examined and compared to assess the invariance of SICBS across EBP general and specific EBP referents. Results indicated that the constrained model fit significantly worse than the freely estimated model, ${\chi }^{2}\left(60\right)=84.3, p< .05$. Conversely, other fit indices less sensitive to sample size improved from the freely estimated to fully constrained model (∆TLI = 0.01, ∆CFI = 0.01, ∆RMSEA = −0.05). We examined the *q* and *d*_Cox_ statistics to further probe the potential lack of invariance. The *q* statistic value for all factor loadings clustered around zero (range = −0.05–0.02). The *d*_*Cox*_ statistics were below the 0.50 cutoff for all thresholds (range = 0.00—0.30). Despite the significant ${\chi }^{2}$ difference test, the preponderance of evidence (i.e., improvement in CFI, TLI, and RSMEA; low *q* and *d*_Cox_ statistics) indicate that the SICBS is invariant across general and specific EBP referents.

### Convergent and divergent validity

Table 5 presents bivariate correlations between the adapted SICBS total and subscale scores and measures of convergent (top of the table) and divergent (bottom of the table) validity. Aligned with CFA results, correlations among SICBS subscales (*r*s = 0.78–0.83) and the total score (all *r*s = 0.92) were high. Associations between global OCB and SICBS subscale and total scores (convergent validity) were moderate (*r*s = 0.43–0.49) and higher than those for PTSQ (*r*s = 0.31–0.38; divergent validity), which were weak. Moreover, SICBS total and subscale scores were unassociated with most school-level demographics but were somewhat positively related to attendance rates and negatively related to the percentage of non-White students in the school.

**Table 5:** Correlations among theoretically related and unrelated variables

		**SICBS**^**1**^				
**Convergent**	**SICBS**^**1**^	A	B	C	D	E
	A. Helping Others	1.00				
	B. Keeping Informed	.828^**^	1.00			
	C. Taking Initiative	.781^**^	.783^**^	1.00		
	D. Advocacy	.787^**^	.789^**^	.819^**^	1.00	
	E. Total	.921^**^	.924^**^	.918^**^	.921^**^	1.00
	**OCBS**^**2**^					
	Total	.429^**^	.430^**^	.466^**^	.480^**^	.490^**^
**Divergent**	**PSTQ**^**3**^					
	Total	.314^**^	.367^**^	.349^**^	.380^**^	.383^**^
	**School Demographics**					
	School Size	.040	.087	.114^*^	.163^**^	.110^*^
	% White	.064	.097^*^	.072	.096	.090
	% Non-White	-.115^*^	-.148^**^	-.148^**^	-.175^**^	-.160^**^
	% Transitional Bilingual	-.052	-.080	-.021	-.065	-.060
	% Special Education	-.011	-.071	-.048	-.048	-.049
	% Attendance Rates	.102^*^	.146^**^	.094	.133^**^	.130^*^

^1^School Implementation Citizenship Behavior Scale

^2^Organizational Citizenship Behavior Scale

^3^Public School Teacher Questionnaire

^*^Correlation is significant at the 0.05 level (2-tailed)

^**^Correlation is significant at the.01 level (2-tailed)

## Discussion

This study examined the psychometric properties of the School Implementation Citizenship Behavior Scale (SICBS) among general education teachers’ implementing universal school-based prevention programs. Three key findings emerged. First, taking initiative and advocacy were confirmed as new subscales related to, yet statistically distinct from, the two original ICBS subscales (helping others, keeping informed). Second, results supported the structural, convergent, and divergent validity of the instrument. Third, the SICBS was found to be invariant across versions of the survey with EBP-specific or EBP-general referents. The ability to robustly measure ICB presents an opportunity to both leverage and track implementer behaviors likely to enrich the overall quality of EBP implementation within an organization.

The two new SICBS subscales reflect the idea that different behavioral approaches may be needed to overcome the unique collection of implementation barriers that characterize specific service settings [37]. Schools that lack implementation support staff often struggle to successfully implement schoolwide prevention programs like SWPBIS [21]. Teachers who take initiative (e.g., voluntarily take on additional implementation-related responsibilities) may be bridging existing resource gaps to facilitate EBP implementation in their school. Moreover, teachers who are vocal about the benefits of EBP (i.e., advocacy) may be setting and maintaining social norms and expectations that help foster enthusiasm around EBP implementation. These types of social norms may be especially important in schools given the effectiveness of universal prevention programming depends largely on high-fidelity implementation across all implementing teachers [49].

 The invariance of the SICBS across EBP referent type is consistent with the School Implementation Leadership [40] and Climate [66] Scales, which are instruments developed alongside SICBS as part of the larger study. Schools commonly implement a multitude of EBPs simultaneously, making instruments that reliably measure ICB irrespective of the EBP referent critical to progressing our understanding of how ICB manifests and exerts influence in schools. The moderate levels of helping others and keeping informed (Table 3) were slightly higher than those reported by school-based mental and behavioral health consultants [37], thoughmore aligned with those reported by mental and behavioral health service providers and their supervisors [19, 63]. Although this might simply be a function of differences across regions and school districts, it also could reflect the strong orientation toward teaming and professional development that tends to characterize many teachers.

### Implications for improving implementation citizenship behavior in schools

EBP implementation is a socially-mediated process that involves working with and through others to create the conditions for initiating, improving, and sustaining behavior change. ICB appears to be an important facilitator of EBP implementation [8, 20, 67] and may prove critical to supporting implementation success in schools. Identifying approaches that enhance ICB in schools, where implementation quality continues to vary substantially [49], may be warranted. Leadership and Organizational Change for Implementation (LOCI [3], is an evidence-based implementation intervention that develops strategic implementation leadership and climate [1, 62, 72], ultimately improving teachers’ ICB [12]. Similarly, Helping Educational Leadership Mobilize evidence (HELM—a recent adaptation of LOCI to promote implementation of universal school-based programs [11, 35]—increased implementation climate and teachers’ ICB [36]. HELM encourages school leaders to establish and use distributed leadership teams such that responsibility for organizational change to promote EBP implementation is shared among key school personnel [15]. Successfully enacting change, however, hinges on identifying appropriate team members—a task for which the pragmatic and psychometrically sound SICBS may be particularly well suited.

### Limitations and future directions

This study provides evidence of the strong psychometric functioning of the SICBS. However, these findings are contextualized by several limitations that can inform future research. First, the RMSEA exceeded the recommended threshold of 0.05, which may indicate model misspecification. However, simulations demonstrate that misalignment between CFI and RMSEA tend to reflect population-level trends as opposed to model misspecification [31]. Replication of this work is needed to determine which phenomenon is at play. Second, data were collected from schools implementing one of two universal prevention programs. Applying the SICBS in the context of other school-based program implementation (e.g., Tier 2) is an important next step for continued measure development and validation. Third, the ICBS was validated previously using leader reports of employees’ [8, 20, 67] and school-based mental health consultants’ self-reported implementation citizenship behavior [39]. Studies that examine and compare SICBS across reporters may illuminate under which conditions each version of the instrument is most applicable. Fourth, a critical next step is to empirically examine the potential antecedents (e.g., implementation leadership and climate, implementer characteristics) and outcomes (e.g., program fidelity) of ICB [3]. Fifth, future work should focus on the relative influence of employees’ ICB within organizations and investigate if the SICBS can be used to identify and leverage teachers who are ready and willing to support EBP implementation in any way they can (e.g., as implementation team members) [38]. Finally, this work should be replicated among more broadly diverse implementers to confirm the strong psychometric functioning of the SICBS.

## Conclusion

This study extended a measure of implementation citizenship behavior to include additional extra-role behaviors thought to substantively influence school-based EBP implementation. Evidence supports the structural, convergent, and divergent validity of the resulting four subscales of the School Implementation Citizenship Behavior Scale (SICBS) in a sample of teachers implementing one of two widely used universal school-based prevention programs. As a pragmatic measure, the SICBS can be used to capitalize on and/or track implementer behaviors likely to enhance the implementation and thus the overall impact of universal prevention programs on students' mental and behavioral health outcomes.

## Supplementary Information

Supplementary Material 1.

## Data Availability

The data sets generated and/or analyzed during the current study are not publicly available but are available from the corresponding author on reasonable request.

## Abbreviations

CFA: Confirmatory factor analysis
CFI: Comparative fit index
EBP: Evidence-based practice
HELM: Helping Educational Leaders Mobilize evidence
ICB: Implementation citizenship behavior
ICBS: Implementation Citizenship Behavior Scale
ICC: Intraclass correlation
LOCI: Leadership and Organizational Change for Implementation
OCB: Organizational citizenship behavior
OCBS: Organizational Citizenship Behavior Scale
PATHS: Promoting Alternative Thinking Strategies
PSTQ: Public School Teacher Questionnaire
RMSEA: Root mean square error of approximation
SICBS: School Implementation Citizenship Behavior Scale
SRMR: Standardized root mean squared error
SWPBIS: Schoolwide Positive Behavior Interventions and Supports
TLI: Tucker-Lewis index
WLSMV: Weighted least squares means and variances
